# Positive and relaxed selection on mitochondrial DNA in parasitic versus predatory mites

**DOI:** 10.1098/rstb.2025.0083

**Published:** 2026-04-02

**Authors:** Justin Havird, Endong Wang, Kendra Zwonitzer, Georgina Aitolo, Xuenong Xu, Bo Zhang

**Affiliations:** 1 Department of Integrative Biology, The University of Texas at Austin, Austin, TX 78712, USA; 2 State Key Laboratory for Biology of Plant Diseases and Insect Pests, Institute of Plant Protection, Chinese Academy of Agricultural Sciences, Beijing 100193, China; 3 Key Laboratory of Natural Enemies Insects, Ministry of Agriculture and Rural Affairs, Beijing 100193, China

**Keywords:** mtDNA, mitochondria, Parasitiformes, selection, adaptation, relaxed selection, evolution, genetics, ecology

## Abstract

Mitochondrial genomes (mtDNA) have distinct evolutionary trajectories owing to their inheritance, ploidy and underlying mutation rates, making them prone to accumulate slightly deleterious mutations. Positive selection on mtDNA has been suggested to be important during adaptation to ‘high-energy’ lifestyles and environments. Disentangling positive versus relaxed selection in molecular mtDNA evolution studies is therefore important, although common metrics such as elevated *d*_N_/*d*_S_ ratios (the ratio of non-synonymous to synonymous substitution rates) can be interpreted as signs of both relaxed purifying selection and positive selection. Here, we examined mtDNA evolution in mites (superorder Parasitiformes) to characterize selection during transitions from a parasitic to a free-living, predatory lifestyle. We predicted energetic demands on predatory mites would be associated with positive selection on mtDNA and that reduced effective population sizes in parasitic lineages would lead to relaxed selection. Using newly sequenced mite mitogenomes, we found a signature of accelerated mtDNA evolution in predatory lineages. Unexpectedly, this is likely due to relaxed, not positive selection on the mtDNA of predatory mites, which is supported by massive, ongoing gene rearrangements in the mtDNA of some predatory lineages (family Phytoseiidae). We discuss why ‘high-energy’ lifestyles are not always associated with adaptive mtDNA evolution.

This article is part of the theme issue ‘Evolutionary genetics of mitochondria: on diverse and common evolutionary constraints across eukarya’.

## 1. Introduction

Mitochondrial DNA (mtDNA) has emerged as a popular genetic marker because of its high abundance relative to nuclear DNA and ease of use owing to ‘universal’ primers that amplify the same region across diverse taxa [1,2]. mtDNA variation among species was also assumed to be largely neutral, because of the hypercritical roles of mitochondrial genes in energy conversion in eukaryotes [3–5]. However, this paradigm has changed, with many case studies convincingly demonstrating that mtDNA variation is linked to environmental adaptation [6–8], approximately 26% of amino acid mtDNA substitutions in animals being identified as driven by adaptive evolution [9], and some people arguing for a general role for positive selection on mitochondrial function during environmental changes [10–12]. Given the wealth of mtDNA sequence data available, the generation of complete mtDNA genomes as a ‘by-product’ of many next-generation sequencing approaches, and the functional importance of mitochondria, it has become tempting to use mtDNA variation to test almost any adaptive hypothesis [13].

Although mtDNA sequences remain popular in evolutionary studies, it is important to remember that mtDNA evolves in fundamentally different ways compared with nuclear DNA. mtDNA does not benefit from sexual recombination, is effectively haploid, and is uniparentally inherited in most eukaryotes. Theory predicts these factors should result in a lower effective population size (*N*_e_) for mtDNA relative to nuclear DNA, making mtDNA especially prone to inefficient selection and mutation accumulation [14–16]. The presence of multiple mtDNAs per nuclear genome in many eukaryotes can also create the opportunity for intracellular conflict and ‘selfish’ evolution, where particular mtDNAs increase in frequency over time, despite lowering organismal fitness [17–19]. Mutation rates are also often elevated in mtDNA compared with nuclear DNA in bilaterian animals [20]. Given these features, mtDNA might be especially prone to relaxed selection and the accumulation of deleterious mutations [13,21].

mtDNA is now paradoxically predicted to play a role in environmental adaptation through positive selection of beneficial mutations, while simultaneously being predisposed to accumulate deleterious mutations via relaxed selection. Many studies have used the *d*_N_/*d*_S_ ratio (the ratio of non-synonymous to synonymous substitution rates) to examine signatures of selection on mtDNA [13]. While positive selection for beneficial variation can result in high *d*_N_/*d*_S_ ratios, relaxed selection against deleterious variation can also cause high *d*_N_/*d*_S_ ratios. Authors often interpret high *d*_N_/*d*_S_ ratios based on previous hypotheses, with studies that explicitly disentangle relaxed versus positive selection being rare. For example, high *d*_N_/*d*_S_ ratios in primate mtDNA relative to other mammals were interpreted as positive selection for energetic efficiency associated with bigger brains (the ‘brain-energy hypothesis’) [22–24], while high *d*_N_/*d*_S_ ratios in flightless birds’ and flightless insects’ mtDNA were interpreted as relaxed selection due to reduced energetic needs [25,26]. Importantly, some approaches can explicitly separate relaxed from intensified positive selection. Chak *et al.* [27] and Maeda *et al.* [28] used RELAX [29] to show that high *d*_N_/*d*_S_ ratios were associated with relaxed, not positive selection in the mtDNA of eusocial shrimps and bivalves with doubly uniparental inheritance, respectively. Recently, we found that when explicit tests are used, high *d*_N_/*d*_S_ ratios in mtDNA are often not caused by the types of selection originally hypothesized [13]. For example, we found little support for relaxed selection in mtDNA of flightless birds or insects and convincing evidence of relaxed, not positive selection in mtDNA of primates [13]. Therefore, it is important to carefully interpret evolutionary signatures in mtDNA and test for alternative hypotheses.

The shift from a free-living to a parasitic lifestyle is often correlated with changes in molecular evolution. For example, transitions to parasitism may be associated with decreases in *N*_e_ due to reliance on a host [30], leading to faster rates of molecular evolution, accumulation of deleterious mutations,, and less efficient environmental selection in parasites. While parasitism has independently evolved hundreds of times across the tree of life [31,32], the shift to an obligate parasitic lifestyle in microbial endosymbionts exemplifies this trend, with many cases of extreme gene loss, gene rearrangements, and greatly accelerated molecular evolution in endosymbionts compared with their free-living relatives [33–35]. Additionally, positive selection on individual genes may also characterize parasitic transitions by allowing parasites to exploit their hosts more effectively. The resulting evolutionary arms races between parasites and hosts are well documented. Studies looking into mtDNA evolution in parasitic lineages have generally mirrored results from the nuclear genome: parasitic lineages often show elevated rates of mtDNA evolution, highly rearranged mitogenomes, loss of conserved mitochondrial genes, or loss of the mitogenome or organelle altogether [36–44]. In a recent study, parasitism explained approximately 45% of the variation in mtDNA evolutionary rates of bilaterians, with rates of evolution being highest in endoparasites, followed by ectoparasites, and then free-living species [41]. However, mtDNA has also been hypothesized to be under positive selection in parasitic wasps [45].

Mites (subclass Acari) have undergone multiple transitions to and from parasitism, including ecto- and endoparasitism [46]. Mites in the family Phytoseiidae are not parasites, but predators with a ‘high-energy’ lifestyle and four distinct types of diets [47]. Briefly, within the superorder Parasitiformes, ticks (families Ixodidae and Argasidae) have low locomotor capacity, as their legs are specialized to cling to hosts (although we note they can still move freely and have higher locomotory abilities than endoparasites) [48,49]. However, predatory mites within Phytoseiidae and Macrochelidae are fast-moving and free-living cosmopolitan predators, preying on arthropods and nematodes that live on plants and decaying litter [50,51]. Other families within Parasitiformes show a mix of predatory and parasitic lifestyles, with parasites generally being adapted to feed on hosts and showing a lower reliance on locomotion overall during their life cycles [52]. Here, we investigated signatures of selection on the mtDNA of mites, especially focusing on phytoseiids, using new mitogenomes. We tested two mutually exclusive hypotheses stemming from general observations in bilaterian animals [41]: (i) parasitic mites have increased rates of mtDNA evolution owing to relaxed selection, or (ii) predatory mites have increased rates of mtDNA evolution owing to positive selection for increased locomotor performance. Finally, we asked whether feeding type within phytoseiids (generalist, specialist, selective, and pollen feeding) was correlated with mtDNA evolution.

## 2. Methods

### (a) New and curated mitogenomes

We sequenced four new mitogenomes from predatory Phytoseiidae mites (*Neoseiulus californicus*, *Neoseiulus barkeri*, *Amblyseius eharai* and *Amblyseius orientalis*) and extracted protein-coding genes from another new phytoseiid based on publicly available sequence reads available via the NCBI sequence read archive (SRA; *Phytoseiulus macropilis*, SRR13571394). We also extracted protein-coding genes from another two new parasitic mite mitogenomes from the closely related family Laelapidae (order Mesostigmata) based on SRA sequence reads (*Pneumolaelaps niutirani*, SRR10269756, and *Gromphadorholaelaps schaeferi*, SRR18036333). We note that other mitogenomes for *N. californicus* and *N. barkeri* became available after the completion of this study and were grouped with our sequences in *post hoc* phylogenetic analyses (NCBI GenBank accession numbers OR195436, NC_069213, OQ026345, ON262343 and OR947717).

For the four new mitogenomes, husbandry, DNA extraction, sequencing, and mitogenome assembly followed our previously published methods for phytoseiid mites [53]. Briefly, mites were raised in the laboratory on flour mites [54,55], except for *N. californicus,* which were fed on *Tetranychus urticae* [56]. For each mite species, approximately 500 adults were sampled from isofemale lines and preserved in ethanol, and DNA was extracted using a Qiagen DNeasy Blood and Tissue Kit. Illumina MiSeq libraries were then prepared, and 250 bp paired-end reads were sequenced at Lianchuan Biotechnology (Hangzhou, China). After quality filtering and trimming, de novo genomes were assembled using SPAdes (v. 3.9.0) [57], and resulting contigs were screened as potential mitogenomes by searching with the COX1 protein sequence of *Phytoseiulus persimilis* via BLAST [58]. Mitogenomes were retrieved as single approximately 16 kb contigs and annotated using MITOS webservers [59] and tRNAcan-SE [60] with the invertebrate mitochondrial code. For the three taxa where protein-coding genes were assembled from existing SRA data, Geneious v. 20251.2 (https://www.geneious.com) was used to map SRA reads to the protein-coding genes from a closely related species, and consensus sequences based on mapped reads were extracted.

We curated an additional 51 mite mitogenomes from NCBI’s GenBank database, focusing on phytoseiid mites, their close relatives and related parasitic mites. Briefly, we followed previous phylogenetic inferences [53] to select mites with available mitogenomes that spanned parasitic and predatory lifestyles (electronic supplementary material, table S1). We focused on the order Mesostigmata, which was mostly represented by predatory mites in our dataset, but included representatives from the parasitic order Ixodida and used *Demodex brevis* (from a separate superorder, Acariformes) as an outgroup. We note that some species we classified as parasitic are not technically parasites but are generally closely associated with hosts in commensal relationships and tend to have lower locomotor capacity (electronic supplementary material, table S1).

### (b) Phylogenetic inference, ancestral state reconstruction, and mitochondrial DNA rearrangements

Complete coding sequences were extracted from the mitogenomes for all 13 protein-coding genes. The amino acid sequences of each gene were aligned using MUSCLE [61] as implemented in MEGA [62] and then back-translated into nucleotide sequences. Custom scripts used to download, parse, align and process mitogenomes are found at https://github.com/thekzwon/mito_accessions_to_full_alignment. All 13 genes were concatenated, and phylogenetic relationships were inferred using this concatenated alignment via maximum likelihood using RAxML v. 8.2.12 [63] with 1000 rapid bootstraps and the PROTGAMMALGF model of evolution, as performed previously [53]. We estimated the ancestral lifestyle (parasitism or predation) at each node of the resulting phylogeny using the *ace* function within the R package *ape* [64]. Lifestyles were coded as discrete characters in terminal taxa, and marginal estimation was used to produce likelihood values of each lifestyle at each node of the phylogeny, along with a model assuming equal rates of transitions between lifestyles.

To characterize gene rearrangements in the mites investigated here, we used CREx [65] as implemented on the EU Galaxy webserver [66] to calculate the number of breakpoints in mtDNA gene order between each pair of taxa. We also compared observed gene orders with the hypothesized ancestral mtDNA gene order for arthropods based on *Limulus polyphemus* [67]. As done previously [53], we excluded *Metaseiulus occidentalis* and *Euseius nicholsi* from this analysis owing to gene duplications in these taxa. We also excluded new mtDNAs based on SRA data because gene order information could not be inferred. Finally, we performed gene rearrangement analyses when considering all mtDNA genes (13 protein-coding genes, 22 tRNAs and 2 ribosomal RNAs (rRNAs)) and only considering the 13 protein-coding genes because tRNAs can show rapid rearrangements and misannotations.

### (c) Tests of selection

We first calculated the *d*_N_/*d*_S_ ratio, a measure of non-synonymous substitution bias, using the codeml function in PAML v. 4.8 [68] along with the phylogeny generated via RAxML. We used two different models in PAML: the first applied a common *d*_N_/*d*_S_ ratio across all branches in the phylogeny (‘model = 0’), while the second applied two different *d*_N_/*d*_S_ ratios (‘model = 2’) to branches leading to parasitic versus predatory mites. Any internal branches were coded based on the lifestyles of representative terminal branches or were coded as parasitic when either state could have been ancestral (although coding ambiguous internal branches in different ways produced qualitatively similar results). The fit of the two models was compared using a likelihood ratio test. This analysis was performed for both the concatenated set of all 13 protein-coding genes and for each protein-coding gene individually.

Elevated *d*_N_/*d*_S_ ratios can be caused by intensified positive selection or relaxed purifying selection. To distinguish between these causes, we explicitly tested for intensified versus relaxed selection on predatory compared with parasitic mite mitogenomes using RELAX [29] as implemented on the Datamonkey Webserver [69]. RELAX uses an input phylogeny divided into two sets of branches: ‘test’ and ‘reference’ branches. It then examines the distribution of *d*_N_/*d*_S_ ratios in each set of branches based on three categories of *d*_N_/*d*_S_ ratios. If *d*_N_/*d*_S_ ratios tend to converge towards 1 in the test branches, then selection is relatively relaxed in these branches, resulting in a *k* statistic <1. If *d*_N_/*d*_S_ ratios diverge from 1, then *k* > 1 and intensified selection is assumed. The significance of the *k* parameter is assessed by comparison via a likelihood ratio test with a model where the same *d*_N_/*d*_S_ categories are applied across all branches. In our analyses, branches leading to predatory mites (and consensus internal branches) were labelled as ‘test’ branches, with branches leading to parasitic mites labelled as ‘reference’ branches, as in PAML analyses. RELAX analyses were also performed for all genes individually and the set of 13 concatenated genes.

We also investigated *d*_N_/*d*_S_ ratios and signatures of relaxed selection on predatory mites with different feeding types. Here, we followed the designation of feeding types of phytoseiid predatory mites by McMurtry *et al.* [47]: type I—highly specialist predators on *Tetranychus* spp. (*Phytoseiulus* spp. in our dataset); type II—broadly specific on *Tetranychus* spp., but includes other prey (*N. californicus, Neoseiulus womersleyi* and *M. occidentalis* in our dataset); type III—generalist predators (*A. eharai, A. orientalis, Amblyseius swirskii, Amblyseius tsugawai, N. barkeri* and *Neoseiulus cucumeris* in our dataset); and type IV—species for which pollen constitutes an important part of the diet (*E. nicholsi* in our dataset). Here, we ran one PAML model with six different branch types (using ‘model = 2’), one branch type for each of the four feeding types in phytoseiid mites (and their consensus internal branches), one branch type for predatory mites outside of Phytoseiidae, and one branch type for parasitic mites. We then ran four iterations of RELAX, where branches leading to each feeding type were iteratively selected as ‘test’ branches and all other branches in Phytoseiidae were set to ‘reference’ branches (other branches were left unclassified). For the analyses on feeding types, only the set of 13 concatenated mtDNA genes was used.

## 3. Results

**Figure 1.**
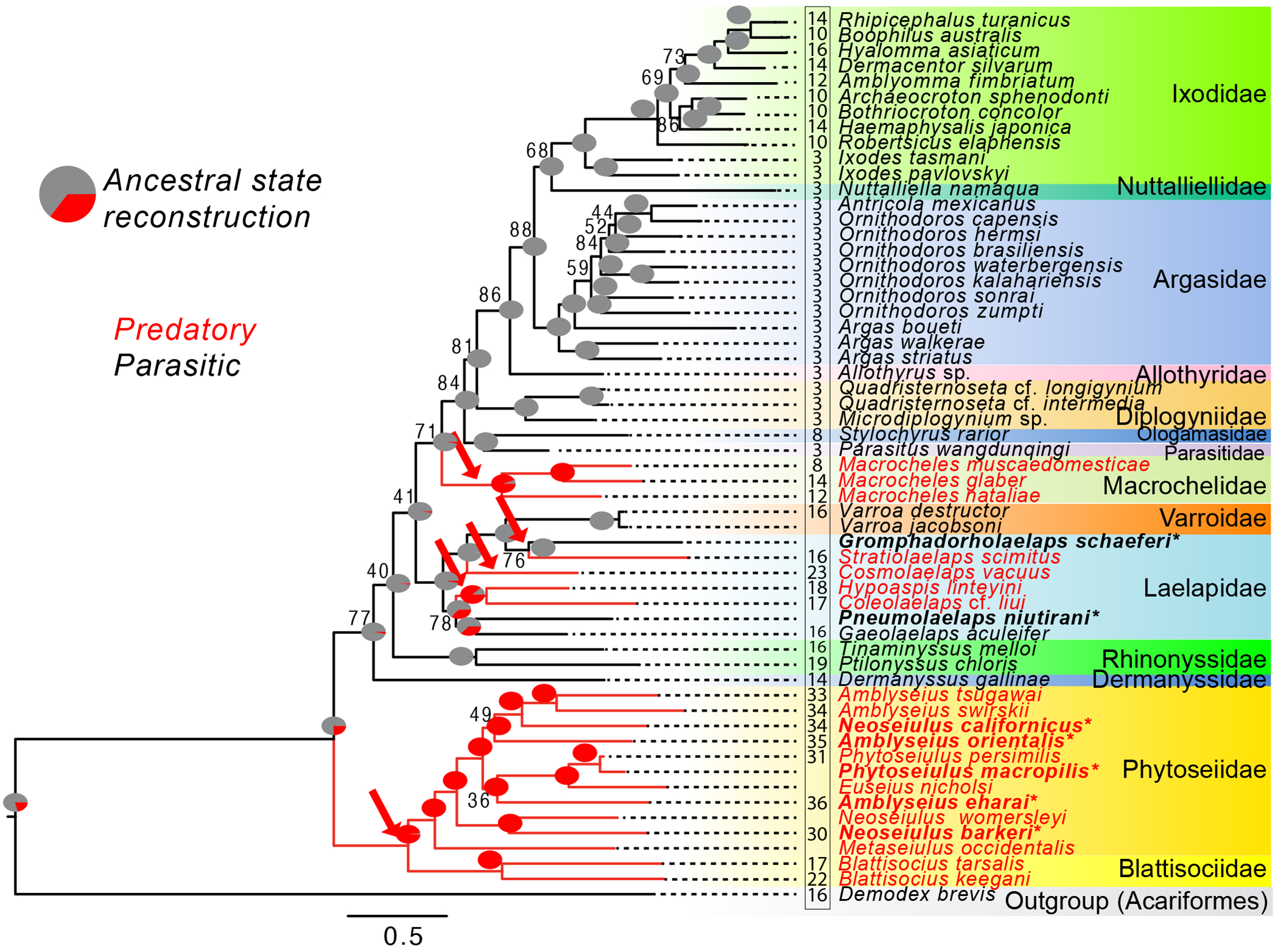
Phylogenetic relatedness among parasitiform mites with parasitic or predatory lifestyles based on concatenated protein-coding genes from mitogenomes. Predatory taxa branches and names are in red, while parasitic ones are in black (with branch colour indicating coding in PAML and RELAX analyses). Pie charts at each node show the likelihood of predatory versus parasitic lifestyles based on ancestral state reconstruction. The five most likely independent origins of predation are indicated with arrows. Taxa in bold with asterisks represent newly generated mitochondrial DNAs reported here. Numbers in the box are pairwise breakpoint distances for gene rearrangements between taxa and the likely ancestral mtDNA gene order in arthropods (see electronic supplementary material, table S2). Bootstrap support values are indicated at nodes, except for nodes with >95% support. Scale bar indicates number of amino acid replacements per site.

Recovered relationships based on mitogenomes were largely as expected and were similar to previous mtDNA phylogenies emphasizing phytoseiid mites, suggesting several independent origins of a predatory lifestyle [53] (figure 1). Specifically, we find strong support for a monophyletic Phytoseiidae and its sister relationship with Blattisociidae, although genera within Phytoseiidae were generally not monophyletic (e.g. *Amblyseius* and *Neoseiulus*), as suggested in a recent phylogenomic analysis of Phytoseiidae [70]. Other families were also generally recovered as monophyletic with moderate to strong support. However, the relationships among some families were different from our previous study [53]. Notably, Mesostigmata is paraphyletic in our current analysis, as the last common ancestor of all Mesostigmata families also includes the orders Holothyrida and Ixodida as descendants. However, the support values for relationships among the other Mesostigmata families are fairly weak, and support for a monophyletic Ixodida is strong. We attribute the differences in the relationships recovered here to a different set of taxa sampled (including different numbers of outgroup taxa) and different phylogenetic methods (which included both maximum likelihood and Bayesian phylogenetics previously). Importantly, these differences do not affect the conclusion that predatory lifestyles arose independently in parasitiform mites. The most likely scenario suggests five independent origins of predation based on ancestral state reconstruction in the current dataset. However, we note that a single origin followed by multiple losses within the Laelapidae may be equally parsimonious.

In general, breakpoint analyses revealed that gene rearrangements were higher in phytoseiids compared with the other mites examined here (electronic supplementary material, tables S2 and S3). The number of breakpoints compared to the ancestral arthropod mtDNA gene order averaged 33 ± 1.0 (s.e.m.) in phytoseiids, and only 10 ± 1.0 in non-phytoseiids (figure 1). Breakpoints comparing different species within phytoseiids were also quite numerous (averaging 28.0), suggesting gene rearrangements are ongoing within the family, although we note that the common ancestor of phytoseiids likely had a highly rearranged genome compared to the ancestral arthropod gene order. Excluding phytoseiids, predatory mites still showed higher numbers of breakpoints compared to the ancestral gene order on average than parasitic taxa (averaging 7.8 in parasitic taxa versus 16.3 in non-phytoseiid predatory taxa). However, phylogeny also played a role in this pattern (figure 1). For example, the number of breakpoints was relatively high in the parasitic family Rhinonyssidae (17.5 ± 1.5), but not particularly high within the predatory family Macrochelidae (11.3 ± 1.8). Moreover, within parasitic Ixodidae, *Ixodes* has a low number of breakpoints (3), while the clade containing all other taxa has experienced a transition to a relatively high number of breakpoints (12.2 ± 0.7), despite also being parasitic. This suggests highly rearranged mitogenomes in the taxa examined here may not be caused by a transition to predation. Patterns of gene rearrangements were qualitatively similar using either all mt genes or just protein-coding genes (electronic supplementary material, tables S2 and S3).

**Figure 2.**
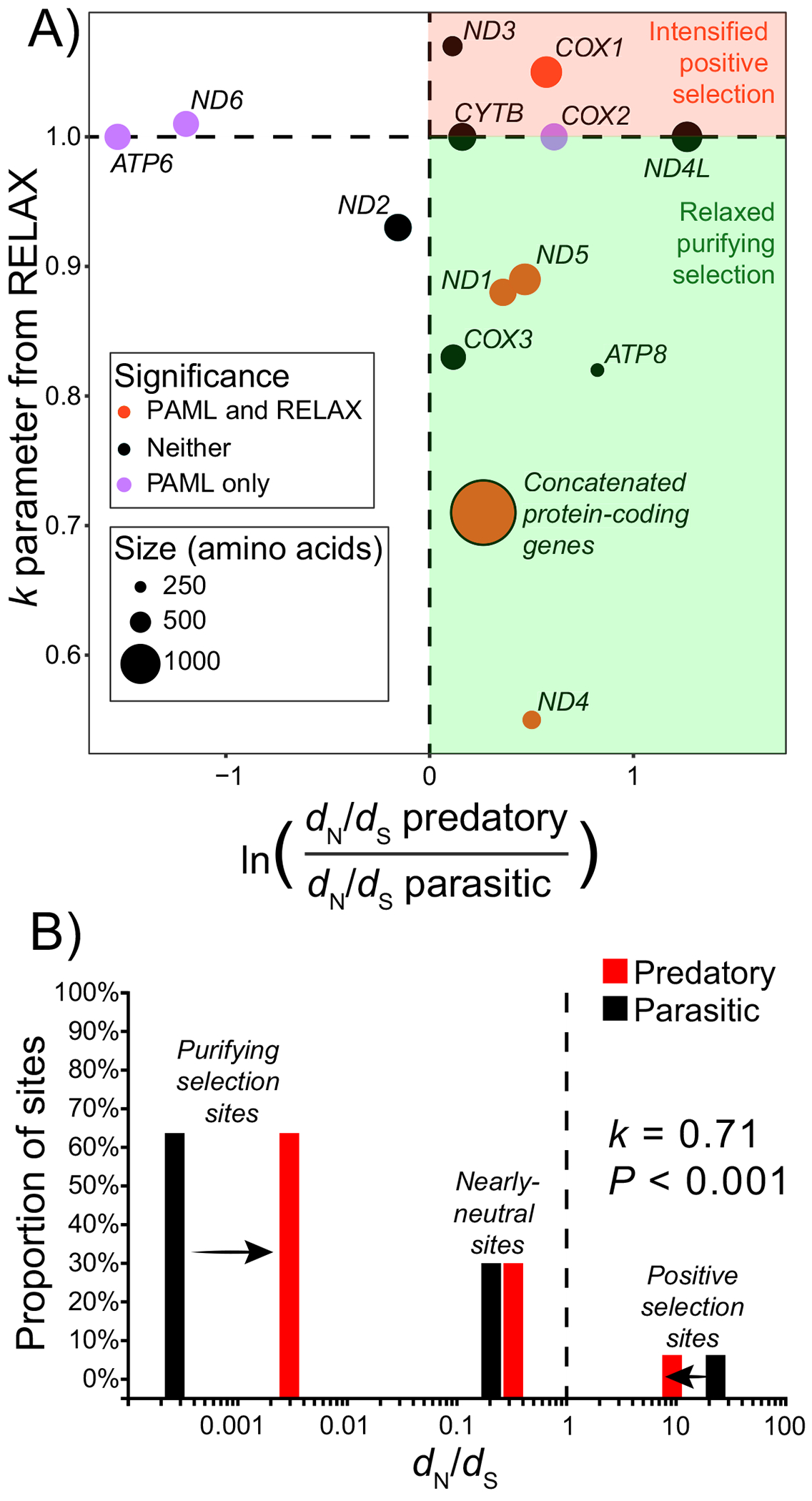
Mitochondrial genes from predatory mites evolve faster than those from parasitic mites, likely owing to relaxed purifying selection. (A) Ratios of non-synonymous to synonymous substitution rates (*d*_N_/*d*_S_) from the two-rate model in PAML analyses are reported on the *x*-axis, while the *k* statistics from RELAX analyses are reported on the *y*-axis for each of the 13 mtDNA protein-coding genes and the concatenated set: *x*-axis values >0 indicate increased *d*_N_/*d*_S_ ratios in predatory mites, while values <0 indicate decreased *d*_N_/*d*_S_ ratios in predatory mites. *y*-axis values >1 indicate intensified positive selection; *y*-axis values <1 indicate relaxed purifying selection on predatory mites. Together, points in the lower right quadrant suggest relaxed purifying selection in mitochondrial DNA (mtDNA) genes of predatory mites, while those in the upper right quadrant suggest intensified positive selection. Each point indicates a single mtDNA protein-coding gene or the concatenated gene set, with point size scaled to the number of amino acid positions in the dataset. Point colour indicates whether models with different rates of evolution were significant for PAML analyses (purple), both PAML and RELAX analyses (orange), or neither (black). (B) RELAX results for the concatenated set of mtDNA protein-coding genes show that branches leading to predatory taxa are evolving under relaxed selection, as *d*_N_/*d*_S_ ratios in the purifying and positive selection classes of sites tend to converge towards 1 relative to the parasitic taxa.

In the concatenated set of 13 mitochondrial genes, *d*_N_/*d*_S_ ratios were significantly elevated in predatory compared with parasitic mites (0.047 versus 0.036, *p* < 0.001 via likelihood ratio test, figure 2A). A similar trend was found in several individual genes, with 10 out of the 13 genes showing higher *d*_N_/*d*_S_ ratios in predatory mites. However, for *ATP6* and *ND6*, *d*_N_/*d*_S_ ratios were significantly lower in predatory mites (by about fourfold each, *p* = 0.002 and 0.018, respectively, figure 2A).

RELAX tests generally suggest that elevated *d*_N_/*d*_S_ ratios in predatory mites were driven by relaxed, not intensified positive selection. In the concatenated set of 13 mitochondrial genes, *d*_N_/*d*_S_ ratios tended to converge on 1 in predatory versus parasitic mites, reflecting relaxed selection (*k* = 0.71, *p* < 0.001, figure 2). This was also the case for three individual genes, *ND1*, *ND4* and *ND5*, where significantly elevated *d*_N_/*d*_S_ ratios in predatory mites were likely driven by relaxed purifying selection (*k* < 0.9, *p* < 0.008 for all, figure 2A). However, there was one gene with elevated *d*_N_/*d*_S_ ratios in predatory mites that was possibly driven by intensified positive selection, *COX1* (*k* = 1.05, *p* = 0.006, figure 2A). For all other individual genes, including *ATP6* and *ND6,* where *d*_N_/*d*_S_ ratios were significantly lower in predatory mites, RELAX analyses did not show significant signatures of relaxed or intensified selection (figure 2A).

For the analyses comparing mtDNA evolution among predatory mites with different feeding types, we generally found elevated *d*_N_/*d*_S_ ratios were confined to the Phytoseiidae+Blattisociidae clade, while other predatory mites showed *d*_N_/*d*_S_ ratios similar to parasitic mites (0.038 versus 0.037). Within Phytoseiidae, type I specialists had the lowest *d*_N_/*d*_S_ ratios (0.044), followed by type III generalists (0.048), type II specialists (0.053) and type IV pollen feeders (0.083). RELAX analyses suggested these elevated *d*_N_/*d*_S_ ratios were generally attributed to relaxed, not intensified selection (0.84 < *k* < 1). However, none of these signals was statistically significant, with the most elevated *d*_N_/*d*_S_ ratio in type IV pollen feeders being borderline (*p* = 0.052). We note that these comparisons have little statistical power, given the low number of branches in our phylogeny leading to any particular feeding type.

## 4. Discussion

Positive selection on mitochondrial genes has become a common finding in evolutionary biology owing to the wealth of mtDNA genetic data available and the relative ease of linking almost any phenotype of interest to ‘energetics’ [12,13,71,72]. However, it is important to consider alternative hypotheses to explain why mtDNA evolution is accelerated in particular lineages, especially when elevated *d*_N_/*d*_S_ ratios are reported across whole mitochondrial genomes and follow-up studies linking mtDNA variation to function or fitness are lacking. Specifically, elevated *d*_N_/*d*_S_ ratios can also be caused by relaxed purifying selection. Disentangling intensified positive selection from relaxed purifying selection is critical; otherwise, studies with opposite results can be used to support the same adaptive storytelling paradigm [73]. For example, elevated *d*_N_/*d*_S_ ratios in island versus mainland birds have been used to argue that island species are prone to less efficient selection owing to lower effective population sizes [74,75]. Paradoxically, when elevated evolutionary rates were reported in mainland species, the cause was attributed to intensified positive selection, not relaxed selection, in mainland species [76]. Therefore, the hypothesis of relaxed selection on islands and intensified selection on the mainland can only be falsified if the causes of elevated *d*_N_/*d*_S_ ratios can be disentangled.

Here, we predicted that the ‘high-energy’ lifestyles of phytoseiid mites would be reflected through intensified positive selection on their mtDNA genes, leading to rapid evolution. If parasitic mites showed faster-evolving mitochondrial genes, we reasoned this would be due to relaxed selection, as suggested from the observation that parasitic lineages with reduced locomotion have elevated mtDNA evolutionary rates across bilaterian animals [41]. Without distinguishing between relaxed and intensified selection as causes of rapid evolution, it would have been easy to accept the overall hypothesis that predatory mites are under stronger selection for ‘energetics’ than their parasitic counterparts, regardless of the results we obtained.

Surprisingly, our results largely support a third scenario: predatory mites have fast-evolving mtDNA genes, but primarily driven by relaxed, not positive selection (figure 2). The concatenated set of mtDNA genes strongly supported this interpretation (figure 2B), along with several individual genes. Only *COX1* yielded a convincing alternative interpretation of higher *d*_N_/*d*_S_ ratios in predatory taxa driven by positive, not relaxed purifying selection (figure 2A). While transitions to a parasitic lifestyle should result in decreased locomotor activity and lower *N*_e_ owing to reliance on a host [30,41], mitogenomic characteristics and life-history traits suggest *N*_e_ may be lower in predatory phytoseiid mites compared with parasitic relatives. While the ancestral mtDNA gene order of arthropods is largely conserved across mites, the family Phytoseiidae shows massive, ongoing gene rearrangements (electronic supplementary material, tables S2 and S3) [53,77]. The new phytoseiid mtDNAs described here exemplify this, with *A. eharai* and *A. orientalis* having 36 and 35 breakpoints compared with the ancestral arthropod mitogenome order. To our knowledge, this meets or exceeds the highest number of breakpoints reported previously at 35 in *Tigriopus japonicus* (Copepoda) and *Heterodoxus macropus* (Hexapoda) [78,79]. This historical and ongoing pattern of gene rearrangements also supports a history of relaxed selection on phytoseiid mtDNA (or lower *N*_e_ and relaxed selection overall), although complete mitogenomes are still limited for this group. Phytoseiids also show marked deviations from parasitic mites in their life-history traits, including faster development times but relatively longer adulthoods, starvation resistance, halting of reproduction when food sources are scarce, haplodiploidy sex determination, and lower census population sizes compared with many parasitic species [80,81]. We also note that most of the parasitic mites included in our dataset are ectoparasites, while elevated rates of mtDNA evolution were most pronounced for endoparasites across bilaterians [41]. Taken together, our results suggest that life-history traits and *N*_e_ may be more powerful in predicting genome-wide mtDNA evolutionary rates than ‘high-energy’ lifestyles.

The choice of dataset and analysis may also heavily influence what factors drive the evolution of mtDNA. We analysed whole mtDNA genes and the concatenated set of mtDNA protein-coding genes for overall elevated *d*_N_/*d*_S_ ratios. Although this is a common approach, it is unlikely that positive selection for increased energetic efficiency would be apparent across the whole mitogenome, or even entire mtDNA genes. OXPHOS and related mitochondrial processes are critical to nearly all eukaryotes that perform aerobic respiration [39], and tend to be under strong purifying selection, even in lineages with ‘low-energy’ lifestyles (e.g. even in plants [82,83]). Indeed, even the signatures of relaxed mtDNA selection reported here for phytoseiids are only relative to parasitic taxa—mtDNA is still under strong purifying selection in predatory mites, and *d*_N_/*d*_S_ ratios were ≪1 for all genes in both parasitic and predatory mites. It is more probable that positive selection would only affect a handful of sites in a specific gene that are critical for specific functions related to different respiratory phenotypes (e.g. excess complex IV capacity or leak respiration). However, we also ran a BUSTED analysis to test for episodic diversifying selection on individual sites in predatory lineages [84] and found no evidence for site-based selection (*p* = 0.18). Despite this negative result, it is still possible that positive selection has characterized mtDNA evolution in predatory mites at some sites/genes, but our evidence suggests that it is not the dominating force driving evolution in the whole mtDNA genome.

More generally, despite mtDNA being inherited as one linked locus, it is unlikely that consistently elevated rates of non-synonymous substitutions across the entire genome are linked to selection for mitochondrial function. We think it is much more likely that such patterns are due to changes in *N*_e_ or other demographic/genetic factors that affect all genes (often including nuclear genes). Coupling patterns across whole mitogenomes with tests to detect selection at specific sites based on *a priori* hypotheses (e.g. [85]) and following up with experiments to test these sequence-based observations may be more appropriate for assessing positive selection on mtDNA.

We also assessed mtDNA evolution in predatory mites with different feeding types, ranging from generalists to different types of specialists. Our results suggest that positive selection on mtDNA genes is not especially intensified in any feeding type but may be especially relaxed in specialists such as pollen feeders. Importantly, these analyses indicated that relaxed selection on mtDNA is not universal across predatory mites but is confined to the Phytoseiidae. This mirrors the finding that phytoseiids, but not necessarily other predatory taxa, have high numbers of gene rearrangements (figure 1) [53].

Taken together, our results suggest that predatory phytoseiid mites show fast rates of mtDNA evolution stemming from relaxed selection, not positive selection related to a ‘high-energy’ lifestyle. We suggest that future studies examining mtDNA evolution in taxa with phenotypes of interest linked with energetics explicitly consider relaxed selection as an alternative hypothesis for fast mtDNA evolution. Further, ‘low-energy’ lifestyles associated with parasitism are not always associated with relaxed selection on mtDNA.

## Supplementary Material

SupplementalTables

Electronic supplementary material is available online at https://doi.org/10.6084/m9.figshare.c.8320525.

## Data Availability

Accession numbers for the sequence data used here can be found in electronic supplementary material, table S1. Supplementary material is available online [86].
